# Evaluation of the Antimicrobial Activity of Chitosan Nanoparticles against *Listeria monocytogenes*

**DOI:** 10.3390/polym15183759

**Published:** 2023-09-14

**Authors:** Sara Pereira, Ana Costa-Ribeiro, Pilar Teixeira, Laura Rodríguez-Lorenzo, Marta Prado, Miguel A. Cerqueira, Alejandro Garrido-Maestu

**Affiliations:** 1 International Iberian Nanotechnology Laboratory, Av. Mestre José Veiga s/n, 4715-330 Braga, Portugal; sara.pereira@inl.int (S.P.); laura.rodriguez-lorenzo@inl.int (L.R.-L.); marta.prado@inl.int (M.P.); miguel.cerqueira@inl.int (M.A.C.); 2 CEB—Centre of Biological Engineering, University of Minho, 4710-057 Braga, Portugal; pilar@ceb.uminho.pt; 3 LABBELS—Associate Laboratory, 4710-057 Braga, Portugal; 4 Department of Biochemistry, Genetics and Immunology, University of Vigo, 36310 Vigo, Spain

**Keywords:** chitosan nanoparticles, antimicrobial, *Listeria monocytogenes*, low molecular weight

## Abstract

Chitosan is obtained from the deacetylation of chitin, and it is known to possess antimicrobial activity. It has attracted attention as it may be used for treating infections caused by different types of microorganisms due to its broad spectrum. Its application in the form of micro- or nanoparticles (CM/CN) has expanded its usage, as in this form, it retains its activity, and remain stable in aqueous solutions. However, inconsistencies in the results reported by different authors have been identified. In this communication, the antimicrobial activity of CN produced from different starting materials was tested against *Listeria monocytogenes*. It was observed that, even though all the starting materials were reported to have a molecular weight (MW) below 200 kDa and degree of deacetylation (DD) > 75%, the size of the CNs were significantly different (263 nm vs. 607 nm). Furthermore, these differences in sizes exerted a direct effect on the antimicrobial properties of the particles, as when testing the ones with the smallest size, i.e., 263 nm, a lower Minimum Inhibitory Concentration (MIC) was achieved, i.e., 0.04 mg/mL. Even though the largest particles, i.e., 607 nm, in individual experiments were able to achieve an MIC of 0.03 mg/mL, the results with CN presented great variation among replicates and up to 0.2 mg/mL were needed in other replicates. The starting material has a critical impact on the properties of the CN, and it must be carefully characterized and selected for the intended application, and MW and DD solely do not fully account for these properties.

## 1. Introduction

Chitosan is a biopolymer that is obtained by the partial deacetylation of chitin, being the second most abundant polysaccharide in nature. Chitin is found as part of the exoskeletons of shrimps, crabs, and lobsters among other sources, and it is an important by-product of the food industry, which is frequently discarded [1,2]. However, chitosan has been reported to have properties including antimicrobial, antioxidant, and antitumor activities that make it attractive for different applications, including its use in the food industry as an additive or for the production of films for food packaging [3]. Finding a second life for these types of discards generated by the food industry to obtain new products, with added value, is of great interest and in line with the Sustainable Development Goals published by the World Health Organization (https://www.who.int/europe/about-us/our-work/sustainable-development-goals).

One of the most commonly explored attributes of chitosan is its antimicrobial activity due to its broad spectrum activity and the emerging problem associated with antimicrobial resistance [4]. It was reported that in chitosan’s pure format the antimicrobial activity is still remarkably lower than that of clinical drugs [1]. However, several parameters are known to affect the antimicrobial properties of chitosan, typically the degree of deacetylation and the molecular weight [5,6], for which low molecular weight chitosan was reported to have a stronger antimicrobial activity [7]. A way to enhance the antimicrobial activity of chitosan is its formulation as micro- and nanoparticles (CM/CN) due to the higher surface-to-volume ratio, allowing for higher interactions with the bacteria compared to the native compound [6,8].

*L. monocytogenes* is a well-known human pathogen, the causative agent of human listeriosis. This microorganism is highly resistant to harsh environmental conditions, and can survive, and even grow, under common refrigeration conditions [9]. Chitosan has the category of GRAS (Generally Regarded As Safe) and is approved as a food additive in several countries [10,11,12]. Furthermore, its application as CM/CN has already been described for the reduction of foodborne bacterial pathogens like *Salmonella* spp. and *Vibrio* spp. in complex matrixes [13,14]. But similar studies targeting *L. monocytogenes* are scarce as most combine the CN with metal ions, essential oils, or other compounds [15,16,17,18], and in most cases, these types of studies are applied for pure cultures [19]. Furthermore, great variation has been observed in the antimicrobial properties reported for CM/CN by different research groups. Thus, the goal of the present study was to determine which chitosan was more suited for the synthesis of CN with antimicrobial activity against *L. monocytogenes* for future applications on food products. To accomplish this goal, in-depth characterization in terms of particle size, zeta potential, Fourier-transform infrared spectroscopy, dynamic light scattering, nanoparticle tracking analysis, and Minimum Inhibitory Concentration was performed, seeking for a relationship between the physicochemical properties and the antimicrobial activity as it is hypothesized that solely the molecular weight of the starting material does not fully account for the antimicrobial activity. 

## 2. Results

### 2.1. CN Synthesis

CNs were synthesized following the synthetic procedure reported by Garrido-Maestu et al. with slight modifications [7]. Two chitosans from different sources, named here as SA1 (Sigma-Aldrich-1, Sigma-Aldrich Co. LLC., St. Louis, MO, USA) and SA2 (Sigma-Aldrich-2, Sigma-Aldrich Co. LLC., St. Louis, MO, USA), were used to create the nanoparticles (NPs) (Table 1 in Materials and Methods section), and the effect of the chitosans’ source on their antimicrobial activity was studied. After CN synthesis, the purification step was modified to improve the CN yield substituting the routine centrifugation by dialysis against ultrapure water. Thus, the final concentration of CN obtained after the production step was 0.02 g/mL for SA1 and 0.017 g/mL for SA2; thus, no major differences in the final concentration of CN generated was observed regardless the starting material selected.

### 2.2. CN Characterization

#### 2.2.1. CN Size and Zeta (ζ) Potential

**Figure 1:**
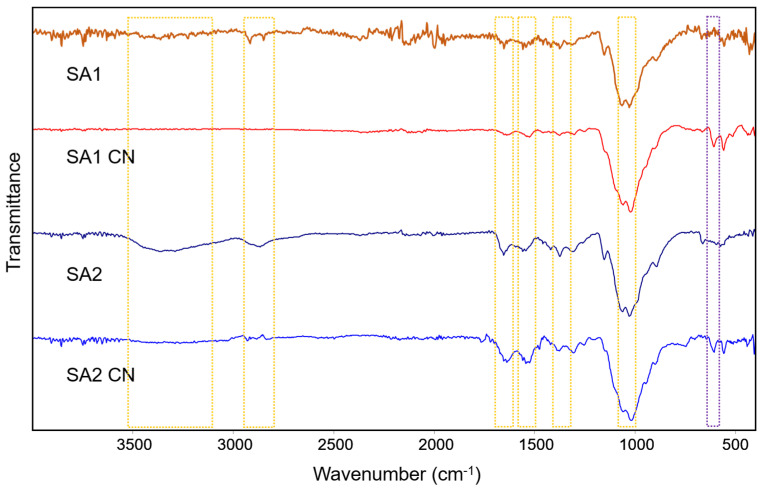
FTIR spectra of chitosan and chitosan NPs. The dotted yellow rectangles indicate the representative bands of chitosan. The dotted purple rectangle indicates the representative band corresponding to sulphate that acts as a cross-linker in the formation of CNs.

CNs were characterized using Fourier-transform infrared spectroscopy (FTIR). Both chitosans alone, and particulate chitosans, showed characteristic bands [21] (Figure 1): ~1025 cm^−1^ and ~1060 cm^−1^ assigned to C-OH stretching vibrations of primary and secondary alcohol, respectively; ~1375 cm^−1^ assigned to C-N bending vibration; and ~1545 cm^−1^ and ~1640 cm^−1^ assigned to N-H bending vibration of N-acetylated residues and C=O stretching of the secondary amide, respectively. Only in the case of SA2 chitosan, the characteristic bands centered at 3360 cm^−1^ and 2865 cm^−1^ for O-H and N-H stretching vibrations and C-H stretching vibration, respectively, were observed. In the case of both SA1 and SA2, the presence of CNs could be observed due to the presence of a characteristic band centered at 612 cm^−1^ that corresponds to the asymmetric bending vibration of sulphate (SO_4_), which acts as a cross-linker in the formation of the nanoparticles [22].

Results also show significant size differences among the CN generated with the different starting materials, even though all of them have low molecular weight. SA1 CN presented the smallest hydrodynamic diameter, while SA2 CN had almost three-fold bigger size (Table 1). This difference can be attributed to a higher contribution, in the case of SA2, in terms of higher weight fraction from highest molecular weight (see Table 1 in Materials and Methods section) [23].

**Figure 2:**
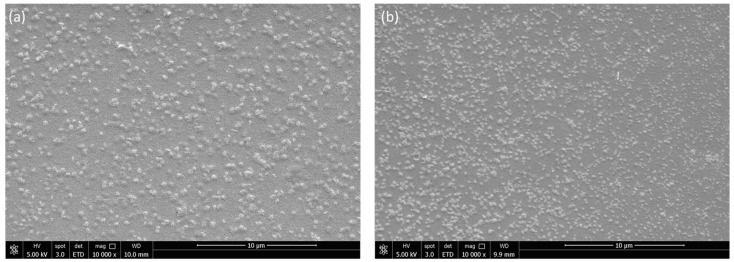
Representative SEM images obtained from SA2 (**a**) and SA1 (**b**) at 10,000×.

Regarding the ζ potential, all the CN presented positive values with an average above 30 mV, except for SA2 which was slightly below this value. Specific data can be found in Table 2. The morphology of the generated CNs was confirmed using Scanning Electron Microscopy (SEM). As it can be observed in Figure 2a,b, the particles generated were spherical. Although the majority of NPs are well dispersed (i.e., single NP), some aggregates are observed in SEM images. This together with the high PDI obtained by DLS proves that these NPs present high polydispersity, i.e., very wide size distribution.

**Figure 3:**
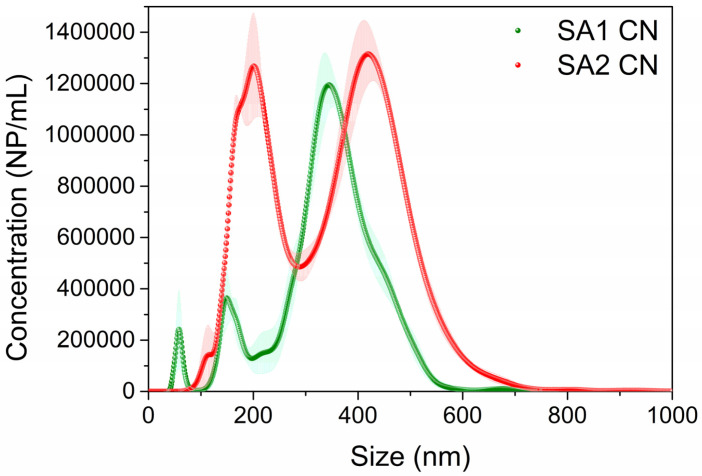
Size distribution of the synthesized CNs obtained using NTA. Color dots and shaded areas show the mean of the NP/mL and the standard error of the mean (SEM), respectively. The samples were diluted 2000× for SA1 CN and 100× for SA2 CN for NTA analysis to achieve <30 NP/frame.

Owing to the high polydispersity observed for both CNs, the hydrodynamic size, size distribution, and NP/mL concentration were measured using nanoparticle tracking analysis (NTA) since it is less sensitive to the presence of different population sizes and the presence of aggregates than DLS [24,25]. Unlike DLS, NTA analysis shows that the average hydrodynamic size of both CNs are similar, only SA1 CN presented slightly smaller size than SA2 CN (see Table 3, mean size and D90). Interestingly, although the mass concentration (g/mL) was similar in both cases (see Section 2.1), SA1 CN displayed a 10-fold higher concentration of NP/mL than SA2 CN: 42 × 10^10^ vs. 4 × 10^10^. This difference can be attributed, in part, to SA1 CN that involves a higher fraction of smaller NPs than SA2 CN, as shown in Figure 3, and the smaller size in terms of mean size, D50 and D90, obtained using NTA (Table 2). However, other possible justification is the stiffness variation between SA1 and SA2, which depends on the electrostatic interactions between the chitosan chains and between chitosan chain and sulphate ions as well as the hydrophobic interactions and hydrogen bond formations between the chitosan chains [26]. Thus, lower stiffness of SA1 could explain the higher number of NPs formed during the synthesis.

#### 2.2.2. Minimum Inhibitory Concentration (MIC)

**Figure 4:**
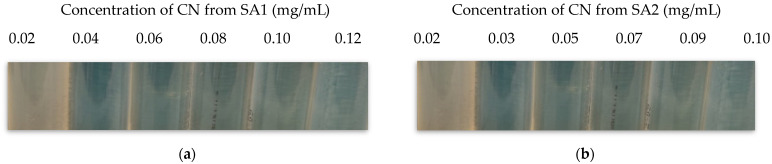
MIC in mg/mL where turbid and clear tubes can be observed for (**a**) SA1 and (**b**) SA2.

The MIC value obtained with *E. coli* O157:H7 was 1.2 mg/mL when using SA1; however, a much lower concentration was determined to be the MIC for this CN when *L. monocytogenes* was used, i.e., 0.04 mg/mL. Regarding the CN generated with the chitosan SA2, the MIC obtained variable results ranging from 0.03 to 0.2 mg/mL (*L. monocytogenes* with CN SA1 was used as a positive control). It is worth noting that this experiment was performed in triplicate and that, regardless of the specific values obtained, only highly reproducible results were obtained with the CN obtained from SA1, while for other materials, a high variability in the MIC values was observed among replicates. A typical outcome of these results is presented in Figure 4a,b where growth inhibition is indicated by the lack of turbidity.

## 3. Discussion

Chitosan is a biopolymer obtained from chitin reported to have a wide range of properties that have attracted the scientific community’s attention. One of these is its antimicrobial activity, which can be enhanced by using chitosan in the form of micro- and nanoparticles (CM/CN). These results are of outmost interest due to the need to identify novel antimicrobials to tackle the increasing threat of antibiotic-resistant microorganisms and, on the other hand, to eliminate microbial pathogens in food products. In this regard, it must be noted that in 2011 an European Regulation was put into place to control the presence of nanomaterials in foods [27]. The antimicrobial activity of CM/CN has been tested against different Gram-negative bacteria, such as *E. coli* and *Salmonella* spp., as well as Gram-positive ones, like *S. aureus* [28,29,30]; however, tests including *L. monocytogenes* are scarce. In addition to this, the antimicrobial properties of CM/CN, as well as native chitosan, present great variability; thus, there is a need for the determination of which starting chitosan material provides best CM/CN in terms of their antimicrobial properties.

It was previously reported that chitosan with low molecular weight allowed for smaller CN to be obtained, and that these exhibited improved antimicrobial activity observed as a lower MIC [7]; thus, in the present study, two different chitosans from a commercial supplier were used for the synthesis of CN. Both presented a similar MW, below <200 KDa, and with similar degrees of deacetylation, >75%. 

The very first interesting observation was that even though both starting materials had similar MW, the CN obtained were remarkably different as only one of the materials allowed to obtain particles with a size below 300 nm, i.e., SA1, and these values were in agreement with previous studies performed with this material; however, the modification in the purification process, namely, the use of dialysis instead of centrifugation, allowed to increase the yield of CN obtained in the original study, from ~20–30% to ~100%, as no CN loses were observed in the dialysis process, while with centrifugation-rinsing protocol, particles were always eliminated in the supernatant [7]. In terms of legislative implications, if intended for food applications, the current CN would be non-compliant to European Regulation 1169/2011 where nanomaterials are defined as those below 100 nm, or larger aggregates but with components below this size, as the individual CN measured in this study were always larger than 200 nm [27]. 

It was observed that the PDI value obtained for these CN, regardless of the starting material, was relatively high, i.e., >0.8. Other studies using the same references and CN synthesis protocols obtained lower values, i.e., 0.3–0.4, with a similar particle size, i.e., 200–500 nm, depending on the specific starting material [7,12]. However, in these studies, the purification procedure consisted of sequential centrifugation steps in comparison to the dialysis performed in the present study. This is also consistent with other studies where using the same CN synthesis protocol, i.e., ion gelation, but with sodium tripolyphosphate as cross-linker, high PDI values were obtained [31,32,33,34]. In addition to this, it must not be overseen that other factors may be involved, for instance, Rodrigues et al. indicated a correlation of the DD with the PDI [33].

In terms of the MIC obtained with each one of the CN, the best results were obtained with SA1 as it reached a value of 0.04 mg/mL, but most importantly, these results were highly reproducible attending based on experiments being performed in triplicate. This is in agreement with the hypothesis that smaller CN may present higher antimicrobial activity due to higher surface-to-volume ratio, thus providing a higher surface for interaction with the microorganisms [7]. In a previous study by Alebouyeh et al., a MIC of 0.8 mg/mL of CN was reported against *L. monocytogenes* [19] being this value significantly higher than those reported in the present study; however, these differences may be explained by the bacterial strain selected, type of chitosan, and the process of generation of CN. All these highlight the relevance of the present work as an attempt to aid in the unification of CN synthesis to obtain comparable antimicrobial activity results among different groups.

In the present study, initially *E. coli* O157:H7 was used as a reference as Garrido-Maestu et al. had already selected this microorganism to evaluate SA1 [7]. It was observed that the MIC obtained with this Gram-negative bacterium was higher than with *L. monocytogenes*, which is Gram-positive. Several studies have reported results in line with ours, where Gram-positive bacteria were more susceptible to CN [19,35]; however, some others have indicated an opposite effect [36,37,38]. As mentioned above, these differences may be associated with not only the MW of the chitosan as typically reported but with the actual source of the material.

Regarding SA2, overall, significantly large particles were generated, i.e., >500 nm, and even though it was possible to reach MIC values in the range of those of SA1, i.e., 0.03–0.06 mg/mL, these results were not reproducible, and up to 0.2 mg/mL was needed in other replicates to inhibit the growth of *L. monocytogenes*. This may be explained by the particle size obtained with this material, as discussed above. It is important to note that even though in the studies of He et al. and Formica et al. a MW of ~200 KDa was reported for this particular chitosan [20,39], Grigoriev et al. indicated that this particular material had a MW of 320 KDa [40], and Garrido-Maestu et al. even indicated a MW of 800 KDa [7]. The discrepancies in the appropriate characterization of the material may have biased our observations as they were based on the initial hypothesis of similar MW and DD. This highlights the importance of proper and accurate characterization of the starting material for the intended application, as shown in this study, and this will have a profound impact on the final result, both in terms of the physicochemical and antimicrobial properties of the CN generated.

## 4. Materials and Methods

### 4.1. Bacterial Strains, Culture Media, and Inoculum Preparation

*L. monocytogenes* WDCM 00021 purchased from the Spanish Type Culture Collection was used as the reference microorganism for the evaluation of the antimicrobial activity of the CN generated. Fresh cultures were prepared by resuspending one single colony in 4 mL of Luria Bertani broth (LB, PanReac AppliChem, Barcelona, Spain) and the suspension was incubated at 37 °C overnight. The following day, the fresh culture was used to prepare a 1:200 dilution in fresh LB and was further incubated at 37 °C under constant agitation, at 120 rpm, until an OD_600_ of ~0.5 was reached (~10^8^ CFU/mL); this new culture was ten-fold serially diluted to reach ~10^6^ CFU/mL, and from this dilution, 100 µL was added to 10 mL of fresh LB (final bacterial concentration of ~10^4^ CFU/mL). This inoculum preparation procedure was followed in order to have the microorganisms under exponential growth rather than in stationary phase.

The same procedure was followed to prepare cultures of *E. coli* O157:H7 AMC 76 kindly provided by the Institute of Applied Microbiology-ASMECRUZ, which was used as control in the determination of the Minimum Inhibitory Concentration (MIC) detailed below.

For both bacterial species, confirmation of the final desired concentration was performed by plating ten-fold serial dilutions of the corresponding cultures on TSYEA and TSA for *L. monocytogenes* and *E. coli*, respectively (both media acquired from Biokar Diagnostics SA, Paris, France). The plates were incubated overnight at 37 °C.

### 4.2. Chitosan Selection, CN Synthesis, and Characterization

#### 4.2.1. Chitosan Selection

A total of three different chitosans, obtained from two different suppliers, were evaluated. All of them had low molecular weight as higher antimicrobial activity was reported compared to medium or high molecular weight alternatives, and with a similar degree of deacetylation. Additional information for each one of the materials tested are provided in Table 2.

#### 4.2.2. CN Synthesis

The generation of the CN was performed following the ion gelation protocol originally described by Jeon et al. and later optimized by Garrido-Maestu et al [7,41]. The protocol was only modified replacing the purification steps originally performed by sequential centrifugation and rinsing with milliQ water, for dialysis. Briefly, the complete protocol consisted of preparing a 2% chitosan suspension in water with 1% Tween 80 (Sigma-Aldrich Co. LLC., St. Louis, MO, USA) and 2% acetic acid (Sigma-Aldrich Co. LLC., St. Louis, MO, USA), and once dissolved, a final concentration of 0.5% of sodium sulfate (Sigma-Aldrich Co. LLC., St. Louis, MO, USA) was added drop-wise under constant stirring and sonication at 60 W for a total of 20 min, and the final CN suspension generated was sonicated for another 25 min. Typically, 100 mL were prepared for each type of starting chitosan. The freshly generated CN was transferred into a dialysis membrane (OrDial D14-MWCO 12000–14000—Orange Scientific, Braine-l’Alleud, Belgium) and placed in a beaker with milliQ water under constant stirring. The water was changed every hour for three consecutive hours, then was left overnight, and on the following day a final water change was performed [7,41,42]. From each CN suspension, the mass concentration was determined using simply weighing. Briefly, 1 mL aliquot of CN sample was taken and dehydrated at 62 °C into a 2 mL glass vial. After drying, the sample-contained vial was weighed and mass corresponded to CN was estimated by the weight difference between empty vial and sample-containing vial. The procedure was conducted in triplicate. 

#### 4.2.3. CN Characterization

##### Attenuated Total Reflectance (ATR) Fourier Transform Infrared Spectrometry (FTIR)

ATR-FTIR analysis was carried out using a Vertex 80v vacuum FTIR Spectrometer (Bruker, Bremen, Germany). Samples were scanned in the wavenumber region from 4000 and 400 cm^−1^. The chitosan sources were analyzed in powder, while 100 µL of CNs was deposited on glass coverslip and dried before the measurement.

##### DLS and ζ Potential

The hydrodynamic size and surface charge of the CNs were estimated using a Nanoparticle Analyzer (SZ-100, Horiba Scientific, Amadora, Portugal). The ζ potential of CN samples was measured in a dispersion of 200 µL/mL in milliQ water at 25 °C. The Smoluchowski approximation [43] was fitted to electrophoretic mobility measurements, and five runs were performed for each sample to calculate the mean and standard deviation (SD). Hydrodynamic size (i.e., Z-average) and polydispersity index (PDI of 200 µL/mL) for aqueous CN dispersions were measured using dynamic light scattering (DLS) at room temperature (i.e., 25 °C) with a scattering angle of 173 °. Five correlation functions were collected over 60 s, each one to estimate the Z-average using the Cumulant method.

##### NTA

The hydrodynamic size, size distribution, and NP/mL concentration were analyzed with NTA using a Nanosight NS300 (Malvern Panalytical, Malvern, UK) provided with a 488 nm CW laser (max. power < 55 mW). The sample was diluted to obtain <30 NP/frame using milli-Q water and 1 mL of diluted sample was placed in a 1 mL plastic syringe (Fisher Scientific, Porto Salvo, Portugal). The analysis was performed using a flow-cell top-plate made of glass and sealed with a PDMS ring, fluxed at a speed of 100, and imaged with a camera level and screen gain set at 13 and 2.1 (slider shutter = 1232; slider gain = 175), respectively. Five videos of 60 s each were recorded (total frames = 1498). The chamber was cleaned after every sample using milli-Q water.

##### Scanning Electron Microscopy (SEM)

SEM analysis of the CN samples were performed in a SEM FEI Quanta 650 FEG (Quanta 650 FEG, FEI, Hillsboro, OR, USA), operating at high vacuum, an acceleration voltage of 5 kV and spot size of three. Five microliters of CN dispersion at a concentration of 50 µL/mL were drop-casted on a silicon wafer and left to dry. The CN-supported Si wafer was placed on pin stub (standard 12.7 mm, 8 mm pin length, Ted Pella Redding, CA, USA), and subsequently, the sample was coated with gold using an EM ACE600 coating system (Leica microsystems Lda, Portugal) to enhance the conductivity of the sample for SEM analysis.

##### MIC

The determination of the MIC of the CN generated with the different starting materials was performed in triplicate covering the range of 0.02 to 2.0 mg/mL using bacterial cultures in exponential phase prepared as detailed in Section 4.1. The bacteria were added to 10 mL of fresh media. The CN were added at fixed volumes, i.e., 10 or 100 µL, and this accounted for the minor differences observed in the final concentrations of the MICs, even though the initial concentration of each material was very similar, i.e., 0.02 vs. 0.017 g/mL, but it was not exactly the same. The tubes with the fresh media, the bacteria, and the CN were incubated horizontally in an orbital incubator (VWR, Radnor, PA, USA) at 37 °C and 120 rpm for 24 h. The assessment was performed by naked-eye observation of turbidity. 

## 5. Conclusions

It was confirmed that the starting material has a critical impact on the final CN obtained; thus, it must be carefully characterized. In this regard, differences in molecular weight and/or degree of deacetylation may not fully account for the differences observed among the CN generated. The effect observed was not only limited by physical properties of the CN such as particle size or ζ potential, but also by other properties like their antimicrobial activity. The data obtained in the current study support previous observations indicating that the CN with the smaller particle size exhibited better antimicrobial properties in terms of consistency of the results, as well as the MIC obtained against *L. monocytogenes*. 

## Figures and Tables

**Table 1:** Chitosan tested in the present study.

Supplier	Reference	MW ^1^	DD ^2^
Sigma-Aldrich-1 (SA1)	448869	50–190	75–85%
Sigma-Aldrich-2 (SA2)	C3646	90–190 *	≥75%

^1^ Molecular weight in KDa. ^2^ Degree of deacetylation. * MW obtained from He et al. [20].

**Table 2:** CN hydrodynamic size, ζ potential, and polydispersity index (PDI).

Chitosan	Size ± SD ^(1)^	ζ Potential ± SD ^(2)^	PDI ± SD ^(1)^
SA1	267.00 ± 53.00	+36.80 ± 14.80	0.83 ± 0.26
SA2	607.00 ± 37.00	+27.03 ± 14.72	0.84 ± 0.18

^(1)^ Hydrodynamic size (i.e., Z-average) measured in nm and polydispersity index (PDI) were estimated using DLS. Five correlation functions were measured at room temperature and scattering angle of 173° for 60 s (mean ± standard deviation (SD)). ^(2)^ ζ potential (mV) was measured five times.

**Table 3:** Parameters obtained using NTA related to particle concentration, particles per frame, mean size, standard deviation, and percentiles of CNs.

	SA1 CN	SA2 CN
NP/mL (×10^10^)	42 ± 8	4 ± 1
NP/frame ^(1)^	19 ± 0.4	25 ± 0.4
Mean size	341 ± 5	350 ± 4
SD	98 ± 5	131 ± 2
Size D10 ^(2)^	194 ± 3	176 ± 3
Size D50 ^(2)^	350 ± 3	366 ± 6
Size D90 ^(2)^	458 ± 7	508 ± 4

^(1)^ The samples were diluted according to ha NP/frame lower than 30 (SA1 was diluted 2000× and SA2 was diluted 100×). ^(2)^ The percentile means are as follows: D90—90% of the total particles are smaller than a particular size; D50—50% of the total particles are smaller than a particular size; and D10—10% of the total particles are smaller than a particular size.

## Data Availability

The data presented in this study are available on request from the corresponding author.

## References

[1] Ke C.L., Deng F.S., Chuang C.Y., Lin C.H. (2021). Antimicrobial Actions and Applications of Chitosan. Polymers.

[2] Divya K., Jisha M.S. (2018). Chitosan Nanoparticles Preparation and Applications. Environ. Chem. Lett..

[3] Wang W., Xue C., Mao X. (2020). Chitosan: Structural Modification, Biological Activity and Application. Int. J. Biol. Macromol..

[4] Ma Z., Garrido-Maestu A., Jeong K.C. (2017). Application, Mode of Action, and in Vivo Activity of Chitosan and Its Micro- and Nanoparticles as Antimicrobial Agents: A Review. Carbohydr. Polym..

[5] Qi L., Xu Z., Jiang X., Hu C., Zou X. (2004). Preparation and Antibacterial Activity of Chitosan Nanoparticles. Carbohydr. Res..

[6] Divya K., Vijayan S., George T.K., Jisha M.S. (2017). Antimicrobial Properties of Chitosan Nanoparticles: Mode of Action and Factors Affecting Activity. Fibers Polym..

[7] Garrido-Maestu A., Ma Z., Paik S.-Y.-R., Chen N., Ko S., Tong Z., Jeong K.C. (2018). Engineering of Chitosan-Derived Nanoparticles to Enhance Antimicrobial Activity against Foodborne Pathogen Escherichia Coli O157:H7. Carbohydr. Polym..

[8] Ma Z., Kang M., Meng S., Tong Z., Yoon S.D., Jang Y., Jeong K.C. (2020). Selective Killing of Shiga Toxin-Producing Escherichia Coli with Antibody-Conjugated Chitosan Nanoparticles in the Gastrointestinal Tract. ACS Appl. Mater. Interfaces.

[9] Allerberger F., Wagner M. (2010). Listeriosis: A Resurgent Foodborne Infection. Clin. Microbiol. Infect..

[10] Kong M., Chen X.G., Xing K., Park H.J. (2010). Antimicrobial Properties of Chitosan and Mode of Action: A State of the Art Review. Int. J. Food Microbiol..

[11] Rampino A., Borgogna M., Blasi P., Bellich B., Cesàro A. (2013). Chitosan Nanoparticles: Preparation, Size Evolution and Stability. Int. J. Pharm..

[12] Ma Z., Garrido-Maestu A., Lee C., Chon J., Jeong D., Yue Y., Sung K., Park Y., Jeong K.C. (2018). Comprehensive in Vitro and in Vivo Risk Assessments of Chitosan Microparticles Using Human Epithelial Cells and Caenorhabditis Elegans. J. Hazard. Mater..

[13] Fan Y., Ginn A., Ma Z., Kang M., Jeong K.C., Wright A.C. (2017). Application of Chitosan Microparticles for Mitigation of Salmonella in Agricultural Water. J. Appl. Microbiol..

[14] Fang L., Wolmarans B., Kang M., Jeong K.C., Wright A.C. (2015). Application of Chitosan Microparticles for Reduction of Vibrio Species in Seawater and Live Oysters (*Crassostrea virginica*). Appl. Environ. Microbiol..

[15] Zimet P., Mombrú Á.W., Faccio R., Brugnini G., Miraballes I., Rufo C., Pardo H. (2018). Optimization and Characterization of Nisin-Loaded Alginate-Chitosan Nanoparticles with Antimicrobial Activity in Lean Beef. Lwt.

[16] Lin L., Gu Y., Cui H. (2019). Moringa Oil/Chitosan Nanoparticles Embedded Gelatin Nanofibers for Food Packaging against Listeria Monocytogenes and Staphylococcus Aureus on Cheese. Food Packag. Shelf Life.

[17] Olaimat A.N., Sawalha A.G.A., Al-Nabulsi A.A., Osaili T., Al-Biss B.A., Ayyash M., Holley R.A. (2022). Chitosan–ZnO Nanocomposite Coating for Inhibition of Listeria Monocytogenes on the Surface and within White Brined Cheese. J. Food Sci..

[18] Milagres de Almeida J., Crippa B.L., Martins Alencar de Souza V.V., Perez Alonso V.P., da Motta Santos Júnior E., Siqueira Franco Picone C., Prata A.S., Cirone Silva N.C. (2023). Antimicrobial Action of Oregano, Thyme, Clove, Cinnamon and Black Pepper Essential Oils Free and Encapsulated against Foodborne Pathogens. Food Control.

[19] Alebouyeh S., Assmar M., Mirpour M. (2020). Effect of Chitosan Nanoparticle from Penaeus Semisulcatus Shrimp on Salmonella Typhi and Listeria Monocytogenes. Iran. J. Public Health.

[20] He Y., Liu C., Xia X., Liu L. (2017). Conformal Microcapsules Encapsulating Microcarrier-L02 Cell Complexes for Treatment of Acetaminophen-Induced Liver Injury in Rats. J. Mater. Chem. B.

[21] Wang A., Li P., Dai Y., Zhang J., Wang A., Wei Q. (2015). Chitosan-Alginate Nanoparticles as a Novel Drug Delivery System for Nifedipine Chitosan-Alginate Nanoparticles as a Novel Drug Delivery System for Nifedipine. Int. J. Biomed. Sci..

[22] Al-Remawi M.M.A. (2012). Properties of Chitosan Nanoparticles Formed Using Sulfate Anions as Crosslinking Bridges. Am. J. Appl. Sci..

[23] Aranda-Barradas M.E., Trejo-López S.E., Real A.D., Álvarez-Almazán S., Méndez-Albores A., García-Tovar C.G., González-Díaz F.R., Miranda-Castro S.P. (2022). Effect of Molecular Weight of Chitosan on the Physicochemical, Morphological, and Biological Properties of Polyplex Nanoparticles Intended for Gene Delivery. Carbohydr. Polym. Technol. Appl..

[24] Caldwell J., Taladriz-Blanco P., Lehner R., Lubskyy A., Ortuso R.D., Rothen-Rutishauser B., Petri-Fink A. (2022). The Micro-, Submicron-, and Nanoplastic Hunt: A Review of Detection Methods for Plastic Particles. Chemosphere.

[25] Filipe V., Hawe A., Jiskoot W. (2010). Critical Evaluation of Nanoparticle Tracking Analysis (NTA) by NanoSight for the Measurement of Nanoparticles and Protein Aggregates. Pharm. Res..

[26] Sacco P., Furlani F., de Marzo G., Marsich E., Paoletti S., Donati I. (2018). Concepts for Developing Physical Gels of Chitosan and of Chitosan Derivatives. Gels.

[27] Commission Regulation (EC) No 1169/2011 Regulation 2011. https://eur-lex.europa.eu/LexUriServ/LexUriServ.do?uri=OJ:L:2011:304:0018:0063:en:PDF.

[28] Veltman B., Harpaz D., Cohen Y., Poverenov E., Eltzov E. (2022). Characterization of the Selective Binding of Modified Chitosan Nanoparticles to Gram-Negative Bacteria Strains. Int. J. Biol. Macromol..

[29] Chandrasekaran M., Kim K.D., Chun S.C. (2020). Antibacterial Activity of Chitosan Nanoparticles: A Review. Processes.

[30] Ngan L.T.K., Wang S.L., Hiep I.M., Luong P.M., Vui N.T., Đinh T.M., Dzung N.A. (2014). Preparation of Chitosan Nanoparticles by Spray Drying, and Their Antibacterial Activity. Res. Chem. Intermed..

[31] Debnath S.K., Saisivam S., Debanth M., Omri A. (2018). Development and Evaluation of Chitosan Nanoparticles Based Dry Powder Inhalation Formulations of Prothionamide. PLoS ONE.

[32] Soltanzadeh M., Peighambardoust S.H., Ghanbarzadeh B., Mohammadi M., Lorenzo J.M. (2021). Chitosan Nanoparticles as a Promising Nanomaterial for Encapsulation of Pomegranate (*Punica granatum L*.) Peel Extract as a Natural Source of Antioxidants. Nanomaterials.

[33] Rodrigues F.C., Devi N.G., Koteshwara K.B., Thakur G. (2020). Investigating the Effect of Chitosan’s Degree of Deacetylation on Size of the Nanoparticle. IOP Conf. Ser. Mater. Sci. Eng..

[34] Sawtarie N., Cai Y., Lapitsky Y. (2017). Preparation of Chitosan/Tripolyphosphate Nanoparticles with Highly Tunable Size and Low Polydispersity. Colloids Surfaces B Biointerfaces.

[35] Chen F., Shi Z., Neoh K.G., Kang E.T. (2009). Antioxidant and Antibacterial Activities of Eugenol and Carvacrol-Grafted Chitosan Nanoparticles. Biotechnol. Bioeng..

[36] Hipalaswins W.M., Balakumaran M.D., Jagadeeswari S. (2016). Synthesis, Characterization, and Antibacterial Activity of Chitosan Nanoparticles and Their Impact on Seed Germination. J. Acad. Ind. Res..

[37] O’Callaghan K.A.M., Kerry J.P. (2016). Preparation of Low- and Medium-Molecular Weight Chitosan Nanoparticles and Their Antimicrobial Evaluation against a Panel of Microorganisms, Including Cheese-Derived Cultures. Food Control.

[38] Dutta J., Tripathi S., Dutta P.K. (2012). Progress in Antimicrobial Activities of Chitin, Chitosan and Its Oligosaccharides: A Systematic Study Needs for Food Applications. Food Sci. Technol. Int..

[39] Formica F.A., Barreto G., Zenobi-Wong M. (2019). Cartilage-Targeting Dexamethasone Prodrugs Increase the Efficacy of Dexamethasone. J. Control. Release.

[40] Grigoriev T.E., Zagoskin Y.D., Belousov S.I., Vasilyev A.V., Bukharova T.B., Leonov G.E., Galitsyna E.V., Goldshtein D.V., Chvalun S.N., Kulakov A.A. (2017). Influence of Molecular Characteristics of Chitosan on Properties of In Situ Formed Scaffolds. Bionanoscience.

[41] Jeon S.J., Oh M., Yeo W., Galvão K.N., Jeong K.C. (2014). Underlying Mechanism of Antimicrobial Activity of Chitosan Microparticles and Implications for the Treatment of Infectious Diseases. PLoS ONE.

[42] Van Der Lubben I.M., Verhoef J.C., Van Aelst A.C., Borchard G., Junginger H.E. (2001). Chitosan Microparticles for Oral Vaccination: Preparation, Characterization and Preliminary in Vivo Uptake Studies in Murine Peyer’s Patches. Biomaterials.

[43] Von Smoluchowski M. (1905). Zur Theorie Der Elektrischen Kataphoress Und Der Oberflachenleitung. Phys. Z..

